# Intersectional inequalities in the health–happiness link via HRQoL across sex, age, and education level

**DOI:** 10.3389/fpubh.2026.1826385

**Published:** 2026-07-14

**Authors:** Byung-Kweon Chang, Soo-Jin Choi, Eui-Jae Lee, Seung-Man Lee

**Affiliations:** 1 Department of Sports Science, Hankyong National University, Anseong, Republic of Korea; 2 Wenzhou Business College Physical Education and Health School, Wenzhou, China; 3 Department of Physical Education, College of Education, Seowon University, Cheongju, Republic of Korea

**Keywords:** community health survey, EQ-5D, happiness, health-related quality of life, physical education, self-rated health

## Abstract

**Background:**

This study explored the associations between health-related quality of life (HRQoL) factors on happiness and self-rated health (SRH). It also identified HRQoL factors and the extent of their association, while providing implications for the direction of physical education.

**Methods:**

This study used the 2022 Korea Community Health Survey conducted by the Korea Disease Control and Prevention Agency. The HRQoL factors examined were athletic ability, self-management, daily activities, pain/discomfort, and anxiety/depression. The dependent variables were happiness and SRH. Correlation and standard multiple regression analysis to analyze the relationship between happiness, health-related quality of life, and self-subjective health.

**Results:**

HRQoL factors demonstrating a significant association with happiness, in descending order, were lower levels of anxiety/depression, lower levels of pain/discomfort, daily activities, and athletic ability. Lower levels of pain/discomfort, athletic ability, daily activities, lower levels of anxiety/depression, and self-management were significantly associated with SRH, in descending order of impact.

## 1 Introduction

Human quality of life is shaped by diverse biological and sociocultural factors, which makes measurement challenging. Nonetheless, several studies have sought to categorize these factors to generate foundational data to support improvement. Emphasis has been placed on health-related factors, given their strong correlation with fundamental human functioning.

A review of previous studies on quality of life revealed diverse research perspectives. Some have investigated the impact of digital artificial intelligence on quality of life (1), while others have examined the effects of vegetarian diets (2). Several approaches have been employed to examine how the concept of quality of life has different meanings across factors such as geographic region (3, 4), age (5, 6), and sex (6, 7).

Health-related quality of life (HRQoL) constitutes a significant portion of quality-of-life research. Widely used indices include the Short Form-36 Health Survey, EuroQol 5-Dimension (EQ-5D), and 5-level EQ-5D (EQ-5D-5L). Previous studies have explored HRQoL in individuals aged 65 years and older (8) and investigated how perceived neighborhood environments affect the quality of life of minority groups in New York (9). During the COVID-19 pandemic, structured reviews assessed the impact of the virus on patients’ HRQoL (10), using both general and disease-specific tools. However, many of these studies have focused on the effects of specific situations rather than evaluating the relative importance or priority of individual quality-of-life factors.

Happiness theory rests on three assumptions: (a) happiness results from comparison; (b) standards of comparison adjust; and (c) standards of comparison are arbitrary constructs (11). Studies on happiness have approached this topic from various perspectives, examining differences based on sex (12, 13) and economic status (14). Research consistently shows that physical activity positively affects happiness (15–17). Even small amounts of physical activity can enhance happiness (18), with individuals across all age groups reporting greater life satisfaction and happiness when they are physically active (19). These findings suggest that physical activity is a key factor in promoting well-being. According to subjective well-being theory, happiness is influenced not only by objective living conditions but also by individuals’ evaluations of their physical and psychological functioning. HRQoL reflects these evaluations by capturing limitations in mobility, self-care, daily activities, pain/discomfort, and emotional well-being. Individuals who experience fewer functional limitations and better psychological health are therefore expected to report higher levels of happiness. Accordingly, HRQoL dimensions can be understood as important determinants of subjective well-being.

Self-rated health (SRH) is a subjective indicator that assesses an individual’s perception of their health status. It has been widely used across various studies due to its simplicity, reliability, and validity. SRH provides a comprehensive evaluation of both physical and mental health, playing a crucial role in predicting long-term health outcomes (20, 21). Some studies have also explored the relationships between sex, age, educational level, and SRH (22, 23).

According to Statistics Korea’s Social Survey, subjective health status is assessed by the proportion of people responding that their health is “very good” or “fairly good.” This measure provides insight into how individuals perceive their physical and mental health, regardless of clinical diagnoses. Between 2010 and 2018, the percentage of people who considered themselves healthy remained stable (46.8 to 48.8%) but is projected to rise to 53.8% in 2024. These findings suggest a growing perception of positive health. However, SRH varies across demographics—men tend to rate their health more positively than women, and younger people generally perceive their health more favorably than older adults. Cultural factors and societal attitudes may also shape SRH assessments. Therefore, further research is necessary on the environmental and social factors that enhance SRH. Self-rated health is widely understood as a global cognitive evaluation of one’s overall health status. Previous research suggests that individuals rely on physical symptoms, functional capacity, and psychological well-being when assessing their own health. Because the EQ-5D dimensions capture these core aspects of functioning and health experience, they are expected to be closely associated with SRH. In particular, mobility limitations, pain/discomfort, and anxiety/depression may directly shape how individuals evaluate their health. Although HRQoL, happiness, and SRH are closely related constructs, they represent distinct dimensions of well-being. HRQoL primarily reflects individuals’ perceived physical, functional, and psychological health status and focuses on health-related aspects of daily life. Happiness refers to a broader evaluation of subjective well-being and life satisfaction that extends beyond health conditions alone. SRH, by contrast, represents an individual’s overall cognitive assessment of their health status and serves as a global indicator integrating physical and mental health perceptions. Thus, HRQoL can be understood as a multidimensional health-related construct, happiness as a broader well-being outcome, and SRH as a summary judgment of perceived health. Clarifying these distinctions is important for understanding how specific HRQoL dimensions may differentially influence happiness and SRH.

SRH is closely tied to environmental conditions. Studies have shown that access to green spaces and public facilities improves individuals’ subjective health (24, 25). Many previous studies have been limited to specific populations, such as certain age groups or educational levels, reducing the generalizability of their findings (26, 27). Large-scale studies involving diverse populations are required to overcome these limitations.

To address these gaps, this study used data from the 2022 Korea Community Health Survey (CHS), which collected data on adults aged 19 and older across South Korea. Unlike prior research, this study focused specifically on health-related factors, regarded as fundamental components of human quality of life. Previous studies have examined the relationships between HRQoL, happiness, and SRH, limited research has comparatively assessed the relative influence of multiple HRQoL dimensions within a unified framework. In addition, many studies have relied on specific or limited samples, restricting generalizability. Accordingly, this study simultaneously examines the effects of multiple HRQoL factors on both happiness and SRH and compares their relative contributions. Furthermore, by situating the analysis within the sociocultural context of South Korea, this study aims to provide a more comprehensive understanding of the relationship between health and well-being. This study makes three contributions to the literature. First, rather than treating HRQoL as a single composite index, it examines the relative influence of individual EQ-5D dimensions on both happiness and SRH, thereby identifying which aspects of health matter most for different well-being outcomes. Second, by analyzing happiness and SRH simultaneously within the same analytical framework, the study clarifies whether the determinants of subjective well-being differ from those of perceived health. Third, using a large nationally representative sample of Korean adults, this study extends previous findings derived from specific populations and provides evidence that is more generalizable at the population level. Together, these contributions advance understanding of the complex relationships between health-related quality of life, subjective well-being, and health perception.

The study aimed to examine the impact of HRQoL factors on happiness and SRH, identify the specific factors and their relative influence, and provide implications for physical education. Building on the literature on subjective well-being and self-rated health, this study proposes that different dimensions of HRQoL may contribute to happiness and SRH through distinct mechanisms. Happiness is generally influenced by both physical functioning and psychological well-being, whereas SRH reflects individuals’ overall evaluations of their physical and mental health status. Accordingly, limitations in mobility, self-care, usual activities, pain/discomfort, and anxiety/depression are expected to reduce both happiness and SRH.

Previous studies have consistently shown that physical functioning, the ability to perform daily activities, freedom from pain, and positive mental health are associated with greater well-being and more favorable health perceptions. Among these dimensions, anxiety/depression may be particularly important for happiness because emotional well-being constitutes a core component of subjective happiness. In contrast, pain/discomfort and mobility may play a stronger role in SRH because they directly affect individuals’ perceptions of their physical health status.

Based on these theoretical considerations, the following hypotheses were developed:

*H1*: Better HRQoL dimensions (mobility, self-care, usual activities, lower pain/discomfort, and lower anxiety/depression) will be positively associated with happiness.

*H2*: Better HRQoL dimensions (mobility, self-care, usual activities, lower pain/discomfort, and lower anxiety/depression) will be positively associated with self-rated health (SRH).

*H3*: Anxiety/depression will show the strongest association with happiness among the HRQoL dimensions.

*H4:* Pain/discomfort and mobility will show stronger associations with SRH than the other HRQoL dimensions.

## 2 Materials and methods

The 2022 CHS used in this study was conducted nationwide by local health centers under the leadership of the Korea Disease Control and Prevention Agency (KDCA). The survey aimed to establish and evaluate regional healthcare plans, standardize the survey implementation system, and produce regional health statistics (28). The Korea CHS has been conducted annually since 2008, and raw data are available on its official website^1^. Among the variables examined in this study, SRH was assessed annually, whereas HRQoL indices and happiness were assessed biannually. Therefore, the 2022 survey provided the most recent data on these variables. The 2024 survey results were released in the first half of 2025, after this study was conducted.

### 2.1 Participants

The study sample comprises adults aged 19 years and older, as defined by South Korea’s resident registration system. The sample was selected using a two-stage sampling process. First, probability-proportional systematic sampling was employed, considering the number of households by housing type at each sampling point. Next, systematic sampling was performed to identify the number of households at each sampling point. A total of 231,785 survey responses were collected from the final sample (See Table 1).

**Table 1:** Participants’ general characteristics.

Variable		Category											
		*N* (%)											
Sex		Male						Female					
		106,119 (45.8)						125,666 (54.2)					
Age (years)		19–29		30–39		40–49		50–59		60–69		>70	
		22,778 (9.8)		24,806 (10.7)		34,351 (14.8)		43,366 (18.7)		50,548 (21.8)		55,936 (24.1)	
Educational level		Less than elementary school				Elementary school graduate				High school or less		College degree or higher	
		11,373 (4.9)				36,584 (15.8)				93,577 (40.4)		90,251 (38.9)	
Health- related quality of Life (EQ-5D)	Athletic ability	I have no difficulty walking.			I have some difficulty walking.			I have to lie down all day.			Refuse to respond		Do not know
		194,187 (83.8)			36,132 (15.6)			1,466 (0.6)			0 (0.0)		0 (0.0)
	Self-management	I have no difficulty bathing or dressing.			I have some difficulty bathing or dressing on my own.			I am unable to bathe or dress independently.			Refuse to respond		Do not know
		218,649 (94.3)			11,543 (5.0)			1,591 (0.7)			0 (0.0)		2 (0.0)
	Daily activities	I have no difficulty performing daily activities.			I have some difficulty performing daily activities.			I am unable to perform daily activities.			Refuse to respond		Do not know
		203,463 (87.8)			26,062 (11.2)			2,254 (1.0)			0 (0.0)		6 (0.0)
	Pain/discomfort	I have no pain or discomfort.			I have some pain or discomfort.			I have severe pain or discomfort.			Refuse to respond		Do not know
		156,486 (67.5)			68,712 (29.6)			6,586 (2.8)			0 (0.0)		1 (0.0)
	Anxiety/depression	I am neither anxious nor depressed.			I am somewhat anxious or depressed.			I am severely anxious or depressed.			Refuse to respond		Do not know
		202,847 (87.5)			26,969 (11.6)			7,948 (0.8)			2 (0.0)		19 (0.0)
Happiness		1 Very dissatisfied	2	3	4	5	6	7	8	9	10 Satisfied	Refuse to respond	Do not know
		1,834 (0.8)	1,538 (0.7)	4,970 (2.1)	38,603 (16.7)	38,603 (16.7)	31,053 (13.4)	49,568 (21.4)	55,776 (24.1)	22,877 (9.9)	18,799 (8.1)	52 (0.0)	241 (0.1)
Self-rated health		Very good	Good		Average		Poor	Very poor		Refuse to respond		Do not know	
		14,425 (6.2)	78,731 (34.0)		95,467 (41.2)		34,827 (15.0)	8,330 (3.6)		0 (0.0)		5 (0.0)	

### 2.2 Items and measurements

The survey was conducted from August 16 to October 31, 2022 (28) using a computer-assisted personal interviewing method, in which trained interviewers conducted one-on-one interviews with households selected as the sample. Telephone verification was conducted with 13% of the respondents who completed the survey to verify the data and ensure quality control. Discrepancies found during this verification process were reported to the KDCA, and the survey data were continuously managed. Ethical approval was not required because the CHS raw data did not include any private identifiers. According to Article 2, Paragraph 2 of the Enforcement Rules of the Bioethics and Safety Act in South Korea, the CHS is not classified as human subject research; it is, therefore, exempt from Institutional Review Board review. All researchers completed training provided by the Korea National Institute for Bioethics Policy in conducting ethical research involving human subjects.

#### 2.2.1 Health-related quality of life (EQ-5D)

In this study, HRQoL (EQ-5D) was used as a concept encompassing athletic ability, self-management, daily activities, pain/discomfort, and anxiety/depression. Each sub-dimension was assessed by asking, “Please select the one option that best describes your health status today.” HRQoL was operationalized in two ways. First, an overall HRQoL index was calculated using the EQ-5D-3L value set for South Korea based on the standard scoring algorithm, where a score of 1 indicates full health. Second, the five EQ-5D dimensions were analyzed separately to examine their relative influence on happiness and self-rated health.

The utility score was calculated as follows:

HRQoL = 1 − (0.05 + 0.096 × MO2 + 0.418 × MO3 + 0.046 × SC2 + 0.136 × SC3 + 0.051 × UA2 + 0.208 × UA3 + 0.037 × PD2 + 0.151 × PD3 + 0.043 × AD2 + 0.158 × AD3 + 0.05 × N3).

#### 2.2.2 Athletic ability

Athletic ability (corresponding to mobility in the EQ-5D) consisted of the following categories: (1) “I have no difficulty walking,” (2) “I have some difficulty walking,” and (3) “I have to lie down all day,” For interpretive convenience and to facilitate comparison across sub-dimensions, responses were reverse coded so that higher values indicated lower levels of athletic ability.

#### 2.2.3 Self-management

Self-management (corresponding to self-care in the EQ-5D) consisted of the following categories: (1) “I have no difficulty bathing or dressing,” (2)” I have some difficulty bathing or dressing on my own,” and (3) “I am unable to bathe or dress independently,.” For interpretive convenience and to facilitate comparison across sub-dimensions, responses were reverse coded so that higher values indicated lower levels of self-management.

#### 2.2.4 Daily activities

Daily activity (corresponding to usual activities in the EQ-5D) consisted of the following categories: (1) “I have no difficulty performing daily activities,” (2) “I have some difficulty performing daily activities,” and (3) “I am unable to perform my daily activities,” For interpretive convenience and to facilitate comparison across sub-dimensions, responses were reverse coded so that higher values indicated lower levels of daily activity.

#### 2.2.5 Pain/discomfort

The pain/discomfort variable comprised the following categories: (1) “I have no pain or discomfort,” (2) “I have some pain or discomfort,” and (3) “I have severe pain or discomfort,” For interpretive convenience and to facilitate comparison across sub-dimensions, responses were reverse coded so that higher values indicated lower levels of pain/discomfort.

#### 2.2.6 Anxiety/depression

The anxiety/depression variable was comprised of the following categories: (1) “I am neither anxious nor depressed,” (2) “I am somewhat anxious or depressed,” and (3) “I am severely anxious or depressed,” For interpretive convenience and to facilitate comparison across sub-dimensions, responses were reverse coded so that higher values indicated lower levels of anxiety or depression.

#### 2.2.7 Happiness

Happiness was measured to assess respondents’ satisfaction with their lives. Participants were asked to select a number between 1 and 10, where one represented “very dissatisfied” and 10 represented “very satisfied.” The survey question used for this measurement was, “Considering all aspects, how satisfied are you (overall) with your life recently?”

#### 2.2.8 Self-rated health

SRH was assessed using the question “How do you perceive your overall health?” The variable consisted of the following response options: (1) Very good was assigned five points; (2) Good was assigned four points; (3) Average was assigned three points; (4) Poor was assigned two points; and (5) Very poor was assigned one point. Higher average values indicated higher SRH levels.

### 2.3 Data analysis

Various statistical analysis techniques were employed to examine differences in key variables across sex, age, and educational level. First, an independent samples t-test was conducted to assess differences in each variable by sex. To investigate the effect of age (ranging from 19 to 106 years) on key variables, including happiness, SRH, and HRQoL, simple linear regression analyses were performed. Additionally, to analyze the mean differences in each variable by educational level (1 = Less than elementary school, 2 = Elementary school graduate, 3 = High school or less, 4 = College graduate or higher), one-way analysis of variance (ANOVA) was conducted. The impact of HRQoL factors on happiness and SRH was measured using multiple regression analyses. Twenty-five participants who responded, “Refused to answer” or “Do not know” to the HRQoL survey were excluded. To explore the impact of HRQoL factors on happiness, 277 participants who responded, “Refused to answer” or “Do not know” were excluded from the analysis. To analyze the effect of HRQoL factors on SRH, four participants with responses of “Refused to answer” or “Do not know” were excluded. All statistical analyses were performed using SPSS version 26.0, with a significance level of 0.05. Given the large sample size, minor deviations from normality were not expected to substantially affect the estimation of regression coefficients.

To reduce potential confounding, the regression models were adjusted for sex, age, and educational level, which are known to be associated with HRQoL, happiness, and self-rated health. Happiness was not included as a covariate in the SRH model, and SRH was not included in the happiness model, because both variables represent conceptually related outcomes rather than background confounders. Thus, the analyses were designed to examine the independent associations of the five EQ-5D dimensions with each outcome after accounting for key demographic characteristics.

## 3 Manuscript formatting

### 3.1 Descriptive statistical analysis

Descriptive statistical analyses (mean, standard deviation, skewness, and kurtosis) were conducted for HRQoL, happiness, and SRH (See Table 2). HRQoL was calculated using the EQ-5D-3L value set for South Korea, with a mean of 0.898, a standard deviation of 0.113, a skewness of −4.142, and a kurtosis of 24.278. Happiness was measured on a 10-point scale, with a mean of 6.96, a standard deviation of 1.793, a skewness of −0.446, and a kurtosis of 0.195. SRH was measured on a five-point scale, with a mean of 3.24, a standard deviation of 0.907, a skewness of −0.284, and a kurtosis of −0.092. Although skewness and kurtosis were within commonly cited reference ranges (29, 30), HRQoL measured by EQ-5D may not be normally distributed; therefore, strict normality was not assumed, and the analyses were conducted considering the large sample size.

**Table 2:** Descriptive statistics for each variable.

Variable	Mean	Standard deviation	Skewness	Kurtosis
HRQoL	0.898	0.113	−4.142	24.278
Happiness	6.96	1.793	−0.446	0.195
SRH	3.24	0.907	−0.284	−0.092

### 3.2 Correlation analysis

Correlation analysis indicated that the correlation coefficients between HRQoL and happiness, HRQoL and SRH, and happiness and SRH were 0.304, 0.484, and 0.356, respectively. All variables were positively correlated at a statistically significant level (*p* < 0.01), and no excessively high correlations were observed, suggesting that multicollinearity was not a concern (See Table 3).

**Table 3:** Correlation analysis.

Variable	HRQoL	Happiness	SRH
HRQoL	1.000	—	—
Happiness	0.304	1.000	—
SRH	0.484	0.356	1.000

Tested using descriptive statistics analysis.

### 3.3 Differences in HRQoL, happiness, and SRH by sex

HRQoL was higher in men (M = 0.912 ± 0.098) than in women (M = 0.886 ± 0.123) (*p* < 0.001). Additionally, happiness was higher in men (M = 7.00 ± 1.796) than in women (M = 6.94 ± 1.789) (*p* < 0.001), indicating a significant difference by sex. Finally, SRH was higher in men (M = 3.36 ± 0.900) than in women (M = 3.14 ± 0.901) (*p* < 0.001), also indicating a significant difference by sex (See Table 4).

**Table 4:** Differences in HRQoL, happiness, and SRH by sex.

Variable	Mean		Standard deviation		t	*p*
	Male	Female	Male	Female		
HRQoL	0.912	0.886	0.098	0.123	55.755	< 0.001***
Happiness	7.00	6.94	1.796	1.789	7.681	< 0.001***
SRH	3.36	3.14	0.900	0.901	58.530	< 0.001***

***p < 0.001, tested using an independent variables t-test.

### 3.4 Differences in HRQoL, happiness, and SRH by age

The effect of age on HRQoL was examined next (see Table 5). The regression analysis revealed that the model was significant (*F* = 47,364.019, *p* < 0.001), with an explanatory power of R^2^ = 0.170. These statistics indicate that age accounted for approximately 17.0% of the variance in HRQoL. The standardized regression coefficient for age was *β* = −0.412, with a t-value of – 217.633 and a significance level of *p* < 0.001, confirming that age had a significantly negative effect on HRQoL. The unstandardized coefficient was −0.001, suggesting that HRQoL decreased by 0.001 units for each additional year of age. The Durbin–Watson statistic was 1.785, indicating that there was no issue of autocorrelation in the residuals. These results suggest that HRQoL tends to decline with increasing age.

**Table 5:** Effect of age on HRQoL.

Dependent variable	Independent variable	B	Std. error	β	t	*p*	Statistics
HRQoL	Constant	0.981	0.000		2253.853	0.000***	R = 0.412 R^2^ = 0.170 Adjusted R^2^ = 0.170 F = 47364.019 *p* = 0.000 Durbin–Watson = 1.785
	Age	−0.001	0.000	−0.412	−2017.633	0.000***	

***p < 0.001, tested using regression analysis.

Following this, the effect of age on happiness was examined (see Table 6). The results indicated that the model was statistically significant (*F* = 3,451.232, *p* < 0.001), with an explanatory power of R^2^ = 0.015, meaning that age explained approximately 1.5% of the variance in happiness. The unstandardized coefficient (B) was −0.012, indicating that for each additional year of age, happiness decreased by 0.012 units. The standardized coefficient (*β*) was −0.121, indicating a relatively weak negative relationship between age and happiness. The t-value was −58.747, with a significance level of *p* < 0.001, confirming that the effect was significant. The Durbin–Watson statistic was 1.659, suggesting no serious issues of autocorrelation among the residuals. These results indicate a tendency for happiness to decrease with age.

**Table 6:** Effect of age on happiness.

Dependent variable	Independent variable	Std. error	β	t	*p*	Statistics
Happiness	Constant	0.012	-	623.327	0.000***	R = 0.121 R^2^ = 0.015 Adjusted R^2^ = 0.015 F = 3,451.232 *p* = 0.000 Durbin–Watson = 1.659
	Age	0.001	−0.121	−58.747	0.000***	

***p < 0.001, tested using regression analysis.

Next, the effect of age on SRH was examined (see Table 7). The results of the regression analysis showed that the model was statistically significant (*F* = 43,545.079, *p* < 0.001), with an explanatory power of R^2^ = 0.158, indicating that age explained approximately 15.8% of the variance in SRH. The unstandardized coefficient (B) was −0.021, suggesting that each additional year of age was associated with a 0.021-unit decrease in SRH. The standardized coefficient (*β*) was −0.398, indicating a moderate adverse effect of age. The t-value was −208.675, with a significance level of *p* < 0.001, confirming that the effect was statistically significant. The Durbin–Watson statistic was 1.828, indicating no serious autocorrelation issues among the residuals. These findings suggest that individuals tend to perceive their health more negatively as age increases.

**Table 7:** Effect of age on SRH.

Dependent variable	Independent variable	Std. Error	β	t	*p*	Statistics
SRH	Constant	0.006	-	763.873	0.000***	R = 0.398 R^2^ = 0.158 Adjusted R^2^ = 0.158 F = 43,545.079 *p* = 0.000 Durbin–Watson = 1.828
	Age	0.001	−0.398	−208.675	0.000***	

***p < 0.001, tested using regression analysis.

### 3.5 Differences in HRQoL, happiness, and SRH by educational level

Differences in each variable according to educational level (1 = Less than elementary school, 2 = Elementary school, 3 = High school or less, 4 = College or higher) were examined next. The effect of educational level on HRQoL is shown in Table 8. The results revealed statistically significant differences in HRQoL across educational levels (*F* = 15429.140, *p* < 0.001). Higher mean values indicate a higher perceived level of HRQoL. Among the groups, those with a college degree or higher reported the highest mean HRQoL (M = 0.932, SD = 0.053), followed by the high school or less group (M = 0.907, SD = 0.098), elementary school graduates (M = 0.835, SD = 0.155), and those with less than elementary education (M = 0.754, SD = 0.196). The results of Scheffé’s *post hoc* test indicated that the differences between all groups were statistically significant at the 0.05 level (d > c > b > a), clearly demonstrating a tendency for individuals with higher educational levels to report better HRQoL.

**Table 8:** Differences in HRQoL by educational level.

Variable	Group	M ± SD	F	*p*	Post hoc – Scheffé test
HRQoL	1. Less than elementary school	0.754 ± 0.196	15429.140	0.000***	d > c > b > a
	2. Elementary school graduate	0.835 ± 0.155			
	3. High school or less	0.907 ± 0.098			
	4. College degree or higher	0.932 ± 0.053			

***p < 0.001, tested using one-way ANOVA.

Next, the effect of educational level on happiness was examined (see Table 9). The results revealed statistically significant differences in happiness across levels of education (*F* = 2,767.073, *p* < 0.001). Higher mean scores indicate greater perceived happiness. The group with a college degree or higher reported the highest level of happiness (M = 7.33, SD = 1.58), followed by the high school or less group (M = 6.86, SD = 1.81), the elementary school graduate group (M = 6.57, SD = 1.95), and the less than elementary school group (M = 6.20, SD = 2.05). According to the Scheffé *post hoc* test, all pairwise mean differences were statistically significant at the 0.05 level (d > c > b > a), indicating a clear trend of individuals with higher educational attainment tending to report greater levels of happiness.

**Table 9:** Differences in happiness by educational level.

Variable	Group	M ± SD	F	*p*	Post hoc – Scheffé test
Happiness	1. Less than elementary school	6.20 ± 2.05	2,767.073	0.000***	d > c > b > a
	2. Elementary school graduate	6.57 ± 1.95			
	3. High school or less	6.86 ± 1.81			
	4. College degree or higher	7.33 ± 1.58			

***p < 0.001, tested using one-way ANOVA.

Following this, the effect of educational level on SRH was examined (see Table 10). The analysis of differences in reverse-coded SRH across educational levels revealed statistically significant results (*F* = 13,348.067, *p* < 0.001). In this context, lower mean scores indicate more positive perceptions of one’s health. The group with a college degree or higher reported the lowest mean score (M = 3.58, SD = 0.78), indicating the most positive perception of their health status, followed by the high school or less group (M = 3.23, SD = 0.85), the elementary school group (M = 2.73, SD = 0.92), and the less than elementary school group (M = 2.39, SD = 0.93). According to Scheffé’s *post hoc* test, all differences between the groups were statistically significant at the 0.05 level (d > c > b > a), indicating a clear tendency for individuals with higher educational attainment to perceive their health more positively.

**Table 10:** Differences in SRH by educational level.

Variable	Group	M ± SD	F	*p*	Post hoc – Scheffé test
SRH	1. Less than elementary school	2.39 ± 0.93	13,348.067	0.000***	d > c > b > a
	2. Elementary school graduate	2.73 ± 0.92			
	3. High school or less	3.23 ± 0.85			
	4. College degree or higher	3.58 ± 0.78			

***p < 0.001, tested using one-way ANOVA.

### 3.6 Effect of HRQol on happiness

A multiple regression analysis was conducted to examine the impact of HRQoL factors on happiness (See Table 11). The results of the regression analysis showed that the model was statistically significant (*F* = 8509.168, *p* < 0.001) and explained approximately 15.5% of the variance in happiness (R^2^ = 0.155, adjusted R^2^ = 0.155). The Durbin–Watson statistic was 1.678, close to 2, indicating no significant autocorrelation in the residuals. All Variance Inflation Factor (VIF) values were below 10, indicating no concerns about multicollinearity.

**Table 11:** Effect of HRQoL on happiness.

Dependent variable	Independent variable	B	S. E.	β	t	*p*	VIF
Happiness	(Constant)	0.360	0.042	—	8.636	0.000***	—
	Athletic ability	0.230	0.014	0.050	16.544	0.000***	2.509
	Self-management	0.030	0.017	0.004	1.733	0.083	1.841
	Daily activities	0.338	0.016	0.069	20.860	0.000***	2.986
	Pain/discomfort	0.265	0.008	0.079	33.247	0.000***	1.544
	Anxiety/depression	1.462	0.010	0.296	143.266	0.000***	1.173

F = 8509.168 (*p* < 0.001), R^2^ = 0.155, adjR^2^ = 0.155, D-W = 1.678. ^***^p < 0.001, tested using multiple regression analysis (MRA).

Regarding the significance of the regression coefficients, athletic ability (*β* = 0.050, *p* < 0.001), daily activities (β = 0.069, *p* < 0.001), pain/discomfort (β = 0.079, *p* < 0.001), and anxiety/depression (β = 0.296, *p* < 0.001) had a statistically significant positive effect on happiness. These findings indicate that happiness increases as athletic ability, daily activities, pain/discomfort, and anxiety/depression improve. However, self-management was not significantly related to happiness. Among the HRQoL factors, anxiety/depression had the strongest impact on happiness (*β* = 0.296), followed by pain/discomfort (*β* = 0.079), daily activities (*β* = 0.069), and athletic ability (*β* = 0.050).

### 3.7 Effect of HRQoL on SRH

Multiple regression analysis was conducted to examine the impact of HRQoL factors on SRH (See Table 12). The regression analysis results showed that the model was statistically significant (*F* = 18,544.700, *p* < 0.001), confirming a meaningful relationship between the independent variables and SRH. The model explained approximately 28.6% of the variance in SRH (R^2^ = 0.286, adjusted R^2^ = 0.286). The Durbin–Watson statistic was 1.817, which was close to 2, indicating that there were no significant autocorrelation issues in the residuals. All VIF values were below 10, confirming that there were no multicollinearity concerns in the model.

**Table 12:** Effect of HRQoL on SRH.

Dependent variable	Independent variable	B	S. E.	β	t	*p*	VIF
SRH	(Constant)	−0.989	0.019		−51.209	0.000***	
	Athletic ability	0.421	0.006	0.181	65.060	0.000***	2.513
	Self-management	0.090	0.008	0.027	11.270	0.000***	1.845
	Daily activities	0.323	0.008	0.130	42.818	0.000***	2.993
	Pain/discomfort	0.400	0.004	0.235	107.753	0.000***	1.545
	Anxiety/depression	0.276	0.005	0.111	58.200	0.000***	1.174

*F* = 18544.700 (*p* < 0.001), R^2^ = 0.286, adjR^2^ = 0.286, D-W = 1.817. ^***^p < 0.001, tested using multiple regression analysis (MRA).

The significance test of the regression coefficients revealed that athletic ability (*β* = 0.181, *p* < 0.001), self-management (β = 0.027, *p* < 0.001), daily activities (β = 0.130, *p* < 0.001), pain/discomfort (β = 0.235, *p* < 0.001), and anxiety/depression (β = 0.111, *p* < 0.001) all had a significant positive effect on SRH. This indicates that, as athletic ability, self-management, daily activities, pain/discomfort, and anxiety/depression improve, individuals tend to perceive their health more positively. Pain/discomfort had the strongest impact on SRH (β = 0.235), followed by athletic ability (β = 0.181), daily activities (β = 0.130), anxiety/depression (β = 0.111), and self-care (β = 0.027).

## 4 Discussion

### 4.1 Differences in HRQoL, happiness, and SRH by sex

The results of this study showed that men scored higher than women across all variables. First, HRQoL, measured using the EQ-5D-3L utility index, was higher in men than in women. This information aligns with previous research showing that men report a higher quality of life than women (31). However, studies on patients with heart failure found no sex or age differences in HRQoL (6), while research on cancer patients reported no sex differences in sociodemographic factors but did observe differences in psychosocial aspects (7). These findings suggest that sex differences in quality of life vary depending on sub-factors, emphasizing the need for sex- and condition-specific approaches to improving HRQoL.

Second, men reported higher happiness than women. While some studies suggest women are happier, this has been attributed to response bias and differing reporting standards. Montgomery’s (12) study, which used sex-sensitive scales, found that men reported higher happiness, supporting the present findings. Other studies, such as one on pre-service teachers, found higher happiness in women. Both sexs improved their happiness through social skills, such as self-expression; however, men’s happiness was negatively affected by entitlement claims and defensive behaviors, factors not observed in women.

Third, SRH was also higher among men. Similar patterns were found in India, where older men reported better SRH, attributed to women’s lower socioeconomic and health status (32). However, other studies reported no significant sex or age differences (23), while some emphasized age as a stronger predictor (32). These inconsistencies suggest that socioeconomic and individual-level factors influence sex differences in SRH. The present findings support previous research showing higher SRH in men, possibly due to sex-related disparities in social and health conditions (33).

### 4.2 Differences in HRQol, happiness, and SRH by age

The findings revealed a consistent pattern in which age was strongly negatively associated with HRQoL and SRH, while its association with happiness was relatively weaker. As individuals aged, they were more likely to report lower quality of life, diminished happiness, and increased negative self-perceptions of health. Among these outcomes, the impact of age was most pronounced in HRQoL and SRH, suggesting that advancing age is more strongly associated with perceptions of physical and mental health than with emotional well-being.

These findings are consistent with previous studies indicating that older adults tend to experience greater health-related challenges, such as reduced mobility, increased pain or discomfort, and vulnerability to psychological conditions, including anxiety and depression (8, 23, 32). For example, Krawczyk-Suszek and Kleinrok (8) reported lower HRQoL scores across all EQ-5D dimensions among individuals aged 65 and older. Similarly, other studies have shown that SRH tends to decline with age, particularly in the presence of chronic disease, physical limitations, or social isolation (22, 23, 32, 33). Interestingly, while age also had a negative impact on happiness, the effect size was relatively small. This suggests that happiness in older adulthood cannot be explained solely by declines in physical health, but also reflects psychosocial adaptation, such as improved emotional regulation, adjusted expectations, and a greater focus on meaningful relationships. These findings are consistent with research suggesting that happiness levels may vary across age groups, with physical activity and life satisfaction playing key roles (18, 19).

Taken together, the findings highlight the need for age-sensitive health promotion strategies. Programs aimed at preserving physical function, preventing pain and discomfort, and managing mental health—especially anxiety and depression—should be prioritized for older adults (20, 21, 25). Additionally, efforts to enhance social connection and emotional well-being can help mitigate age-related declines in happiness and health perception. This study provides evidence supporting the development of life-stage-specific strategies to promote both physical and mental quality of life in aging populations. These findings suggest that physical activity and physical education programs for older adults may help maintain health, happiness, and quality of life.

### 4.3 Differences in HRQol, happiness, and SRH by education level

This study found that educational level was positively associated with HRQoL, happiness, and SRH. Participants with higher levels of education consistently reported higher quality of life, greater happiness, and more favorable perceptions of their health. The clearest pattern emerged in HRQoL and SRH, where those with a college degree or higher significantly outperformed those with lower educational attainment.

These findings are consistent with previous research highlighting the strong link between education and various health outcomes (22, 23, 32). Higher educational attainment is often associated with better health literacy, more proactive health behaviors, access to healthcare resources, and improved socioeconomic status—all of which contribute to higher perceived quality of life and health (29, 33). Additionally, individuals with higher levels of education may have more stable employment, healthier lifestyles, and greater access to supportive social networks, which collectively enhance both physical and psychological well-being.

In terms of happiness, participants with higher educational levels also reported significantly greater life satisfaction. This difference may be due to increased autonomy, income, social mobility, and opportunities for personal development that often accompany higher education. Prior studies have demonstrated that individuals with higher levels of education tend to experience greater happiness, partly due to their increased ability to manage stress and access meaningful life experiences (14, 18).

The consistent positive association between education and all three variables—HRQoL, happiness, and SRH—underscores the importance of addressing educational disparities as part of broader public health strategies. Targeted interventions that promote lifelong learning, adult education, and community health literacy may help narrow these gaps. Moreover, educational policy and physical education programs should work to enhance health awareness and well-being across all educational strata. However, educational level may also reflect differences in income and socioeconomic status. Therefore, the observed associations should not be interpreted as the effect of education alone.

### 4.4 Effect of HRQoL on happiness

Athletic ability positively impacted happiness. However, athletic ability discussed here focuses on basic physical abilities related to walking. Various studies have found that individuals who can perform basic walking activities without difficulty tend to report higher levels of happiness (34, 35). This result is particularly significant for older adults, as walking difficulties increase with age, making mobility a crucial factor in their well-being (35, 36). Performing daily activities can be challenging without sufficient athletic ability. Therefore, physical education programs should focus on maintaining athletic ability, particularly in older adults, rather than primarily targeting younger or middle-aged individuals. Athletic ability in the present study refers to mobility as measured by the EQ-5D. Since this item mainly assesses walking ability, its relevance may differ across age groups. While walking ability is an important indicator of functional health in older adults, it may not fully reflect differences in physical fitness among younger adults.

Daily activities positively influenced happiness. Individuals who performed daily tasks without difficulty experienced higher levels of happiness, whereas those who struggled with daily activities reported lower levels of happiness. This finding aligns with a study by Tadic et al. (37) suggesting that older adults who can manage their daily activities tend to experience greater happiness, whereas those who struggle with daily tasks report lower happiness levels. Several studies have supported the notion that individuals who engage in daily activities experience greater happiness (38, 39). These findings underscore the importance of physical education programs that promote the maintenance of essential physical fitness components.

Lower levels of pain and discomfort were associated with higher levels of happiness. Similar findings have been reported previously. For example, Zwart et al. (40) suggested that individuals who experience pain or a fear of falling tend to report lower happiness levels. Other studies have found that lower levels of pain and discomfort contribute to increased happiness (41, 42). Given that reduced pain and discomfort are associated with higher levels of happiness, it is essential to ensure that physical activity and physical education programs maintain an appropriate exercise intensity to prevent injuries and minimize physical discomfort.

Lower levels of anxiety and depression were associated with higher levels of happiness. Specifically, lower levels of anxiety and depression were associated with increased happiness. Since the 1980s and the early 2000s, numerous studies have explored the relationship between depression and happiness (43, 44). Although physical activity is generally associated with improved mental health, inappropriate intensity or context may lead to adverse psychological responses. Therefore, careful attention should be paid to ensure that physical activity does not have negative psychological effects.

Finally, better self-care was not significantly associated with happiness. In the present study, self-care was measured by bathing and dressing, which should be taken into consideration when interpreting the results. The lack of association between self-care activities and happiness is consistent with prior research. For example, Ibáñez-Rueda et al. (45) found no significant correlation between showering water consumption and life satisfaction, supporting the findings of this study. The type of clothing worn can also influence happiness levels (45, 46). Since bathing and dressing habits may vary by country’s economic conditions, future research should consider cross-national differences when examining the relationship between self-care and happiness.

From a theoretical perspective, the dominant role of anxiety/depression in predicting happiness is consistent with subjective well-being theory, which emphasizes that individuals evaluate their lives not only through objective circumstances but also through emotional experiences. Physical limitations may constrain daily functioning, but psychological distress directly shapes how individuals interpret and evaluate their overall quality of life. This may explain why anxiety/depression emerged as a substantially stronger predictor of happiness than the other HRQoL dimensions. The findings therefore suggest that emotional well-being occupies a central position in the health–happiness relationship and that improvements in physical health alone may not necessarily translate into higher levels of happiness unless accompanied by positive psychological functioning.

### 4.5 Effect of HRQoL factors on SRH

This study found that HRQoL has a significant positive impact on SRH. The factors, ranked in order of influence, were pain/discomfort (*β* = 0.235), athletic ability (β = 0.181), daily activities (β = 0.130), anxiety/depression (β = 0.111), and self-management (β = 0.027). These findings align with previous studies (20, 21, 25), reinforcing that both physical and mental factors play crucial roles in shaping individuals’ health perceptions.

Notably, pain/discomfort was identified as the most influential factor affecting SRH, consistent with prior research indicating that physical discomfort directly affects health perception (20, 25). This finding is particularly relevant for individuals with chronic illnesses or older adults, as physical pain can significantly diminish their quality of life and lead to a lower self-assessment of health (21). Athletic ability was the second most influential factor, highlighting the strong correlation between regular physical activity and health perception, as previously reported (26, 27). These results suggest that individuals who feel physically and mentally energetic are more likely to perceive their health positively, linking this perception to overall health behaviors.

Daily activities also had a relatively strong influence on SRH, demonstrating that an individual’s ability to perform routine physical tasks is a key component of health perception (47, 48). Anxiety and depression also play significant roles in SRH, indicating that mental health is closely tied to overall health perceptions. This finding is consistent with studies showing that individuals with depressive symptoms tend to rate their health more negatively (49–51). Finally, self-management had the lowest influence among the examined factors, yet still had a significant effect on SRH, suggesting that individuals can systematically manage their health, which in turn influences their health perceptions. This finding aligns with previous research that highlights the relationship between self-efficacy and health behaviors (51, 52).

Our findings have several important implications for future studies. First, as physical pain and discomfort were found to have the most significant impact on SRH, it is necessary to strengthen chronic disease management and pain-relief programs when formulating health policies. As societies continue to age, the number of individuals experiencing chronic pain is increasing, highlighting the need for systematic management strategies. Second, as athletic ability and daily activities significantly influence SRH, promoting physical activity and rehabilitation programs is essential. Providing tailored exercise programs for middle-aged and older adults can help shape a more positive perception of personal health. Third, given the significant impact of anxiety and depression on SRH, it is crucial to raise awareness about mental health management and enhance support systems. Expanding mental health counseling and preventive programs, along with efforts to reduce the social stigma surrounding mental health issues, should be prioritized. Fourth, as self-management was found to affect SRH, there is a need to strengthen health education and implement policy measures to encourage individuals to engage in proactive health behaviors.

### 4.6 Overall implications, limitations, and scope for further research

In this study, men scored significantly higher than women in HRQoL, happiness, and SRH, highlighting the importance of both physical and mental factors in health perception. These findings indicate the need for a comprehensive assessment approach tailored to diverse demographic characteristics. Health promotion policies should adopt multidimensional strategies, including chronic pain management, physical activity programs, mental health support, and self-care systems.

Given sex and socioeconomic disparities, enhanced support systems for women and vulnerable groups are essential, alongside strategies that encourage personal health behaviors through self-management education. These efforts could shape future public health policies and inform the direction of physical education. The study also emphasizes the need to shift the traditional focus of physical education from athletic performance to a broader emphasis on health and well-being. Findings showed that pain/discomfort and anxiety/depression significantly impacted happiness and SRH, supporting the integration of health and wellness education within or alongside physical education programs.

Several limitations guide future research directions. The sample was limited to Korean adults aged 19 years and above, which limited the generalizability and the analysis of age-related factors. Future studies should include more diverse populations and age groups. The use of cross-sectional 2022 CHS data limits longitudinal insight, and future research should track changes over time. Additionally, while the large-scale survey offers broad patterns, qualitative methods are necessary for a deeper understanding. Furthermore, although the 2022 dataset was the most recent fully available dataset at the time of analysis, future studies should incorporate more recent data when possible. Finally, the limited number of analyzed variables constrained the discussion, suggesting future studies should explore a wider array of factors. In addition, HRQoL measured using EQ-5D-3L may exhibit skewed distributions and ceiling effects in population-based surveys, which should be considered when interpreting the findings. Moreover, the use of simple linear regression may not fully capture complex relationships, and future studies should consider more advanced analytical approaches. Although the models adjusted for key demographic characteristics, unmeasured socioeconomic factors may have introduced residual confounding. Furthermore, the cross-sectional design precludes causal inference. Future studies should incorporate additional socioeconomic indicators and longitudinal designs.

This study highlights the importance of physical health as well as the need for comprehensive mental health management, underscoring the need to develop systematic health policies and programs in the future. Furthermore, these findings can serve as a foundational resource for public health policymaking and initiatives aimed at improving individual health behaviors.

## 5 Conclusion

Using data from the 2022 CHS, this study analyzed a sample of 231,785 South Korean adults aged 19 years and older to examine the associations between HRQoL factors and happiness and SRH. The objective was to determine the extent to which individual HRQoL factors influence personal health and happiness, and to propose a direction for physical education to enhance well-being. Regarding happiness, lower levels of anxiety and depression showed the strongest association, followed by lower levels of pain and discomfort, fewer problems in daily activities, and greater athletic ability. Self-management was not significantly associated with happiness. Regarding SRH, all five HRQoL factors were significantly associated with SRH. Lower levels of pain and discomfort had the strongest association, followed by greater athletic ability, fewer problems in daily activities, lower levels of anxiety and depression, and self-management.

## Data Availability

Publicly available datasets were analyzed in this study. This data can be found at: can be downloaded after being granted permission, https://chs.kdca.go.kr/chs/rdr/rdrInfoDownMain.do.

## Footnotes

1
https://chs.kdca.go.kr
